# A Support Vector Machine based method to distinguish proteobacterial proteins from eukaryotic plant proteins

**DOI:** 10.1186/1471-2105-13-S15-S9

**Published:** 2012-09-11

**Authors:** Ruchi Verma, Ulrich Melcher

**Affiliations:** 1 Department of Biochemistry and Molecular Biology, Oklahoma State University, Stillwater, OK 74078 USA

**Keywords:** proteobacteria, plant proteins, SVM, machine learning, amino acid composition, dipeptide composition

## Abstract

**Background:**

Members of the phylum Proteobacteria are most prominent among bacteria causing plant diseases that result in a diminution of the quantity and quality of food produced by agriculture. To ameliorate these losses, there is a need to identify infections in early stages. Recent developments in next generation nucleic acid sequencing and mass spectrometry open the door to screening plants by the sequences of their macromolecules. Such an approach requires the ability to recognize the organismal origin of unknown DNA or peptide fragments. There are many ways to approach this problem but none have emerged as the best protocol. Here we attempt a systematic way to determine organismal origins of peptides by using a machine learning algorithm. The algorithm that we implement is a Support Vector Machine (SVM).

**Result:**

The amino acid compositions of proteobacterial proteins were found to be different from those of plant proteins. We developed an SVM model based on amino acid and dipeptide compositions to distinguish between a proteobacterial protein and a plant protein. The amino acid composition (AAC) based SVM model had an accuracy of 92.44% with 0.85 Matthews correlation coefficient (MCC) while the dipeptide composition (DC) based SVM model had a maximum accuracy of 94.67% and 0.89 MCC. We also developed SVM models based on a hybrid approach (AAC and DC), which gave a maximum accuracy 94.86% and a 0.90 MCC. The models were tested on unseen or untrained datasets to assess their validity.

**Conclusion:**

The results indicate that the SVM based on the AAC and DC hybrid approach can be used to distinguish proteobacterial from plant protein sequences.

## Background

**Figure 1:**
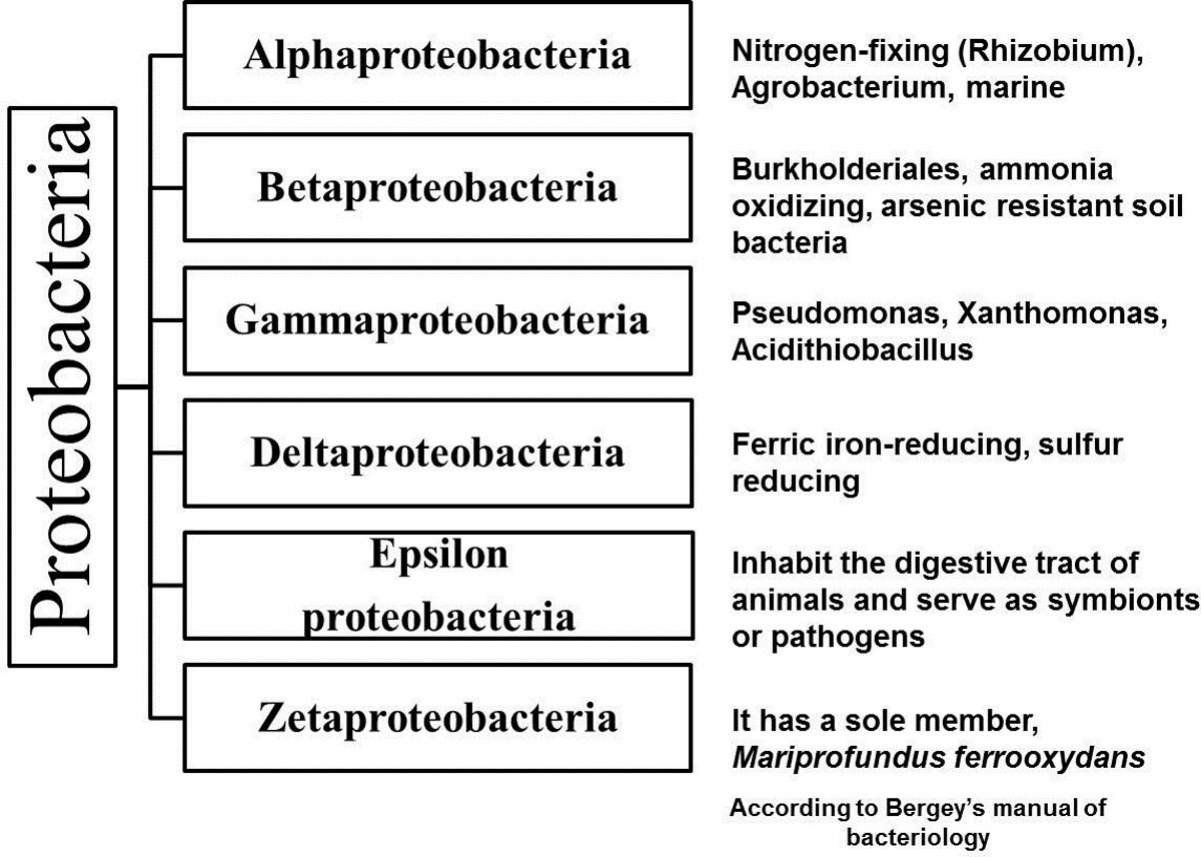
**The subgroups of proteobacteria and the main members of each subgroup**.

Bacterial plant pathogens are a major threat to global food security [1]. Half of the bacterial species causing major food losses in the world belong to the major phylum Proteobacteria (Figure 1). They are found predominantly in the class Gammaproteobacteria (*Xanthomonas, Pseudomonas *and *Erwinia*) and also in the class Betaproteobacteria (*Ralstonia*). Gammaproteobacteria include in addition a wide variety of several medically, ecologically and scientifically important groups such as Enterobacteriaceae (*Escherichia coli*), Vibrionaceae and Pseudomonadaceae. Also, beneficial bacteria, such as nitrogen fixing, ammonia oxidizing and iron fixing bacteria are members of this phylum. Betaproteobacteria also include ammonia oxidizing and arsenic resistant bacteria with Burkholderiales as one of the major classes. Alphaproteobacteria is dominated mostly by nitrogen-fixing bacteria and agrobacteria. Deltaproteobacteria and Epsilonproteobacteria have aerobic genera and curved to spirilloid *Wolinella *spp., respectively. Zetaproteobacteria is composed of a sole member: *Mariprofundus ferrooxydans *which oxidizes ferrous to ferric iron [2].

Several methods are being developed to detect phytopathogens involving macromolecular sequencing, especially nucleotide sequencing [3,4]. With the advent of next generation sequencing, testing of diseased or quarantined plants for the presence of proteobacteria will rely increasingly on massive DNA sequencing. Peptide mass spectroscopy also shows promise in such screening. The analysis of nucleotide sequences typically involves assembly of sequence reads into contigs followed by analysis using Blast [5] search to identify pathogen-derived contigs. This approach is limited in that it only identifies potential pathogens whose nucleotide sequences are included in the searched database. Thus, there is a strong need for methods to find the organismal origin of unknown DNA or peptide fragments to identify potential pathogen sequences. Machine learning techniques, such as support vector machines (SVMs) and neural networks have been used successfully to develop classifiers for a number of different biolgocial problems including predicting different categories of proteins [6-14]. As a first step towards detecting pathogenic bacteria spp., we evaluated whether a machine learning algorithm, SVM, could distinguish between proteobacterial (potential pathogen) and plant (host) proteins. Thus, we assembled datasets of proteobacterial and plant host proteins for this study. We focused on amino acid, rather than nucleotide residues, because of the greater variety of residues that can be present at any one position, allowing subtle evolutionary forces to play a role in shaping the protein sequence and its properties.

## Methods

### Training datasets

**Figure 2:**
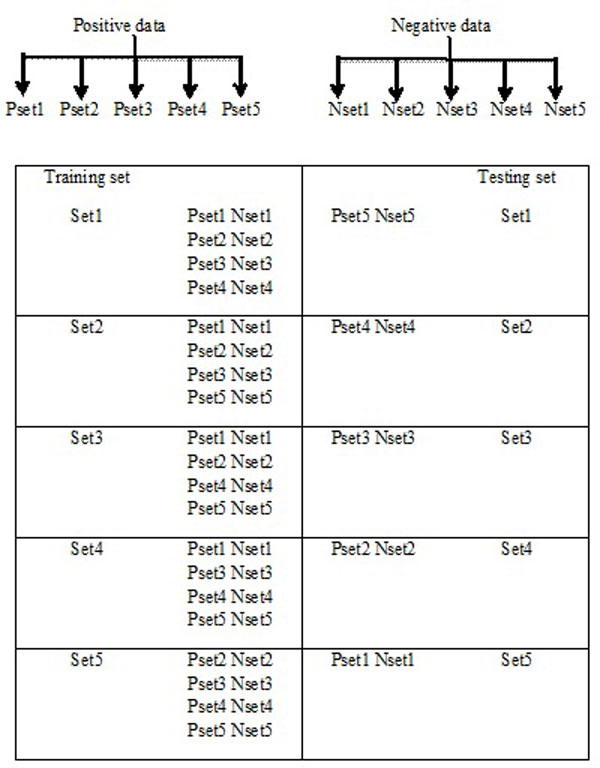
**Construction of datasets using five-fold cross validation**. Pset is for positive dataset (proteobacteria) and Nset is for negative dataset (plants).

Amino acid sequences of proteobacteria and plants were downloaded from the Uniprot website [UniProt release 2012_01-Jan 25, 2012] http://www.uniprot.org/. Only reviewed protein sequences were taken into consideration. A total of 3508 proteins (mean length, 322 ± 202) from nine species of proteobacteria (of which, three are phytopathogens) and 3206 proteins (mean length, 376 ± 308) from ten plant species were used initially for training. We used Blastclust [15] to remove redundant proteins, defined as those having greater than a specified % identity (a % redundancy value) from the data. Redundancy filtering was performed both before and after combining proteins from different species. Datasets were constructed at 90%, 50% and 30% redundancy values. Thus, with the 90% redundancy set we obtained 3408 proteobacterial and 2631 plant host proteins. For the 50% and 30% redundancy sets we obtained 3230 proteobacterial proteins, 2284 plant host proteins and 3203 proteobacterial proteins, 2277 plant host proteins, respectively. As the goal of this study was to identify bacterial proteins, the proteobacterial protein set was taken as the positive class and the plant protein set as the negative class (Tables 1 and 2). Test and training sets were designed from a five-fold cross-validation to create a model for the classification of new sequences (Figure 2). Thus each dataset was in both training and testing sets. To further validate the performance of our best-trained models, we tested the models on unseen/blind or untrained data not used for training the SVM. From Uniprot we downloaded non-redundant proteins for three species of proteobacteria (*Serratia marcesens, Acidovorax citrulli, Rhizobium fredii*) and three plant species (*Solanum lycopersicum, Phaseolus vulgaris, Cucurbita pepo*).

**Table 1:** Total number of pathogen proteins taken from Uniprot and number of proteins remaining after redundancy filtering at 3 different percentages.

Positive dataset (Pathogen)	Total number of proteins (reviewed)	90% redundancy	50% redundancy	30% redundancy
*Agrobacterium tumefaciens (Rhizobium radiobacter)*	104	103	103	103
*Burkholderia phymatum*	333	333	333	333
*Pseudomonas aeruginosa *(ATCC)	1217	1216	1211	1211
*Xanthomonas oryzae pv. Oryzae*	411	410	410	410
*Ralstonia solanacearum*	601	601	599	599
*Rhizobium etli *(ATCC)	424	421	421	421
*Rhizobium meliloti*	48	47	47	47
*Methylobacterium nodulans*	213	213	213	213
*Desulfobacterales autotrophicum *(ATCC)	157	157	157	157
Total	3508	3501	3494	3494
Total after blastclust on cumulative data	-	3408	3230	3203

**Table 2:** Total number of plant proteins taken from Uniprot and number of proteins remaining after redundancy filtering at 3 different percentages.

Negative dataset (Plant host)	Total number of proteins (reviewed)	90% redundancy	50% redundancy	30% redundancy
*Triticum aestivum*	357	315	292	291
*Oryza sativa*	87	86	86	86
*Solanum tuberosum*	390	314	308	308
*Arabidopsis thaliana*	1000	968	857	852
*Cucurbita maxima*	26	25	25	25
*Citrus sinensis*	93	91	91	91
*Vitis vinefera*	161	154	152	152
*Hordeum vulgare*	348	323	307	307
*Pisum sativum*	371	347	335	334
*Glycine max*	373	346	324	324
Total	3206	2969	2777	2770
Total after blastclust on cumulative data	-	2631	2284	2277

### Feature Vectors used

Amino Acid Composition (AAC): Each protein was represented as a vector of 20 features, each corresponding to the fractional composition of an amino acid. This set of feature vectors was presented as input to SVM. Separate amino acid frequencies were calculated for both sets of proteins (proteobacteria and plants). The AAC was calculated by the following equation:

$$\text{Fraction}\;\text{of}\;\text{amino}\;\text{acid}\;\text{x}=\frac{Total\;number\;of\;amino\;acid\;x}{Total\;number\;of\;amino\;acids\;in\;protein}$$

where x can be any amino acid residue.

Dipeptide Composition (DC): Each protein was represented as a vector of 400 features for the 20 × 20 possible combinations of amino acids. The DC was calculated by the following equation:

$$\text{Fraction}\;\text{of}\;\text{dipeptide}\left(\text{xy}\right)=\frac{Total\;number\;of\;dipep\;xy}{Total\;number\;of\;all\;possible\;dipeptides}$$

where dipeptide (xy) is one of 400 possible dipeptides.

Hybrid (AAC+DC): The AAC and DC feature vectors were merged to yield feature vectors of 420 features (20+400).

### Support Vector Machine

**Figure 3:**
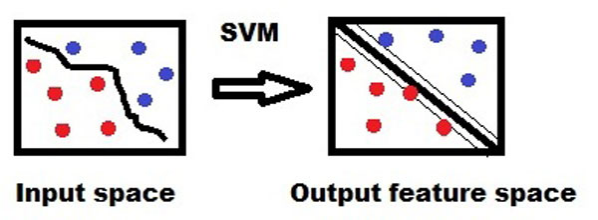
**The concept of Support Vector Machine (SVM) in feature differentiation**.

An SVM is a kernel-based margin classifier, which uses both statistics and optimization. It draws an optimal hyper-plane in a high dimensional feature space that defines a boundary that maximizes the margin between data samples in two classes, therefore giving a better generalization property (Figure 3). Specifically, SVM^light^, which is an implementation (in C language) of SVM, has been used in this study. The SVM^light ^package can be downloaded from http://www.joachims.org for non-commercial or academic use [16]. In this study we used the SVM concept for the classification of proteobacteria and plant (host) proteins. Learning was carried out by using three kinds of kernels: the linear (t = 0), the polynomial (t = 1) and the Radial Basis Function (RBF) (t = 2). We obtained the best performance from the RBF.

### Evaluation

Evaluation of the performance of the three models is threshold dependent. The performance of our method was computed by using the following standard parameters [17,18].

(a) Sensitivity or coverage of positive examples: percent of proteobacterial proteins correctly predicted

$$\text{Sensitivity}\left(\text{Sn}\right)=\frac{\text{TP}}{\text{TP}+\text{FN}}\times 100$$

(b) Specificity or coverage of negative examples: percent of plant proteins correctly predicted as plant protein

$$\text{Specificity}\;\left(\text{Sp}\right)=\frac{\text{TN}}{\text{TN}+\text{FP}}\times 100$$

(c) Accuracy: percent of correctly predicted proteins (bacterial and plant proteins).

$$\text{Accuracy}\;\left(\text{Acc}\right)=\frac{\text{TP + TN}}{\text{TP + FN + FP + FN}}\times 100$$

(d) Matthews correlation coefficient (MCC) is considered to be the most robust parameter of any class prediction method [19]. MCC equal to 1 is regarded as perfect prediction while 0 suggests completely random prediction.

$$\text{MCC}=\frac{\left(\text{TP}\times \text{TN}\right)-\left(\text{FP}\times \text{FN}\right)}{\sqrt{\left(\text{TP + FP}\right)\left(\text{TP + FN}\right)\left(\text{TN + FP}\right)\left(\text{TN + FN}\right)}}$$

where TP represents truly predicted proteobacterial proteins, and TN represents truly predicted plant proteins. FP and FN are falsely predicted proteobacterial and plant proteins, respectively.

## Results and discussion

**Figure 4:**
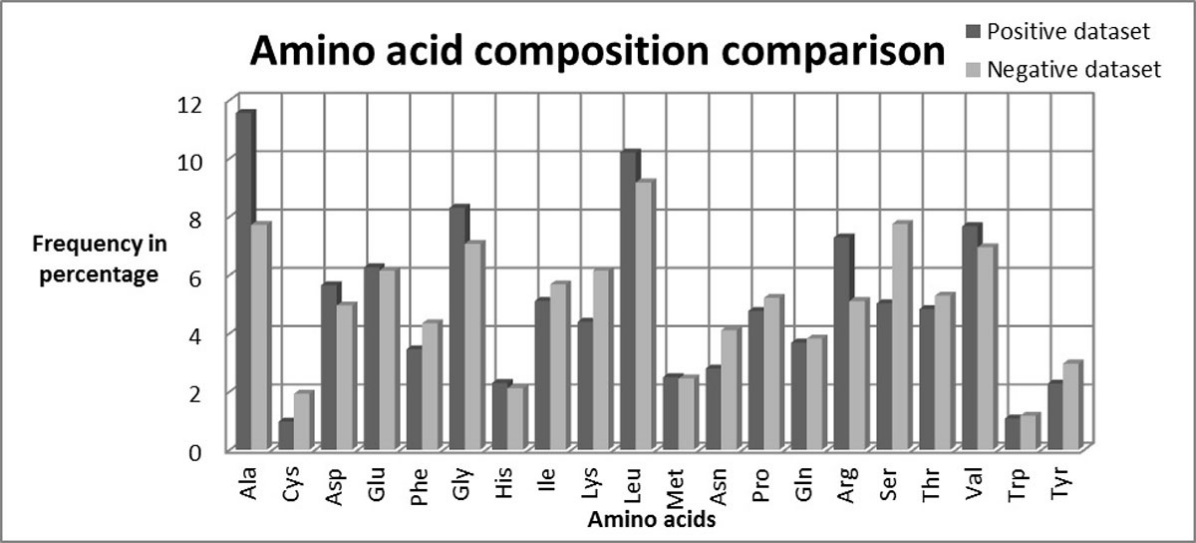
**Comparative amino acid compositions of positive dataset (proteobacteria) and negative dataset (plants)**.

To test whether the AAC of proteobacterial and plant proteins differ significantly, we calculated AAC for both the proteobacterial (Table 1) and plant (Table 2) proteins datasets (Figure 4). We observe differences of AAC between proteobacteria and plants with respect to alanine, cysteine, glycine, lysine, arginine and serine. We also calculated the DC for these two datasets (figure not shown). We input the following vector sets for the SVM: AAC, DC and a hybrid of AAC and DC [20] models. We trained all three kernels (linear (Table 3), polynomial (Table 4) and RBF (Table 5) to identify the best-trained kernel. Comparison of the accuracies and MCCs obtained by all three kernels revealed that the RBF kernel performed best with all three redundancy percentages (Table 5). At 90% redundancy the SVM achieved a maximum accuracy of 92.44% and a 0.85 MCC for the AAC model [RBF parameters: g = 0.04, c = 4, j = 1], a maximum accuracy of 94.67% and 0.89 MCC for the DC model [g = 0.02, c = 6, j = 2] and for the hybrid model a maximum accuracy of 94.86% and a 0.90 MCC [g = 0.01, c = 8, j = 1]. At 50% redundancy, maximum accuracies for the AAC, DC and hybrid models were 91.62% (MCC 0.83) [g = 0.04, c = 2, j = 1], 94.12% (MCC 0.88) [g = 0.02, c = 4, j = 1] and 94.49% (MCC 0.89) [g = 0.01, c = 8, j = 2] respectively. At 30% redundancy, the maximum accuracies of the AAC, DC and hybrid models were 92.30% (MCC 0.84) [g = 0.05, c = 1, j = 1], 93.72% (MCC 0.87) [g = 0.03, c = 2, j = 2] and 93.84% (MCC 0.88) [g = 0.01, c = 4, j = 2]. As shown in Table 5 we achieved maximum accuracy with the hybrid model at 90% redundancy.

**Table 3:** Results of SVM models based on AAC, DC and hybrid (AAC+DC) features at three different redundancy percentages using the linear kernel (t = 0).

Redundancy(percentage)	Amino acid composition (AAC)		Dipeptide composition (DC)		Hybrid (AAC+DC)	
	**Accuracy (%)**	**MCC**	**Accuracy (%)**	**MCC**	**Accuracy (%)**	**MCC**

30	87.87	0.75	90.74	0.81	89.10	0.78
50	87.45	0.74	90.87	0.81	91.81	0.83
90	87.45	0.74	90.87	0.81	89.33	0.78

**Table 4:** Results of SVM models based on AAC, DC and hybrid (AAC+DC) features dataset at three different redundancy percentages using polynomial kernel (t = 1)[d is another parameter used in this kernel and its value is given in parentheses].

Redundancy(percentage)	Amino acid composition (AAC)		Dipeptide composition (DC)		Hybrid (AAC+DC)	
	**Accuracy (%)**	**MCC**	**Accuracy (%)**	**MCC**	**Accuracy (%)**	**MCC**

30	(d5) 89.63	0.78	(d2) 92.06	0.84	(d6) 91.29	0.82
50	(d4) 88.88	0.77	(d2) 91.98	0.83	(d4) 90.68	0.81
90	(d5) 89.46	0.78	(d2) 92.54	0.85	(d4) 91.26	0.82

**Table 5:** Results of SVM models based on AAC, DC and hybrid (AAC+DC) features at three different redundancy percentages using RBF kernel (t = 2)

Redundancy(percentage)	Amino acid composition (AAC)		Dipeptide composition (DC)		Hybrid (AAC+DC)	
	**Accuracy (%)**	**MCC**	**Accuracy (%)**	**MCC**	**Accuracy (%)**	**MCC**

30	92.30	0.84	93.72	0.87	93.84	0.88
50	91.62	0.83	94.12	0.88	94.49	0.89
90	92.44	0.85	94.67	0.89	94.86	0.90

**Figure 5:**
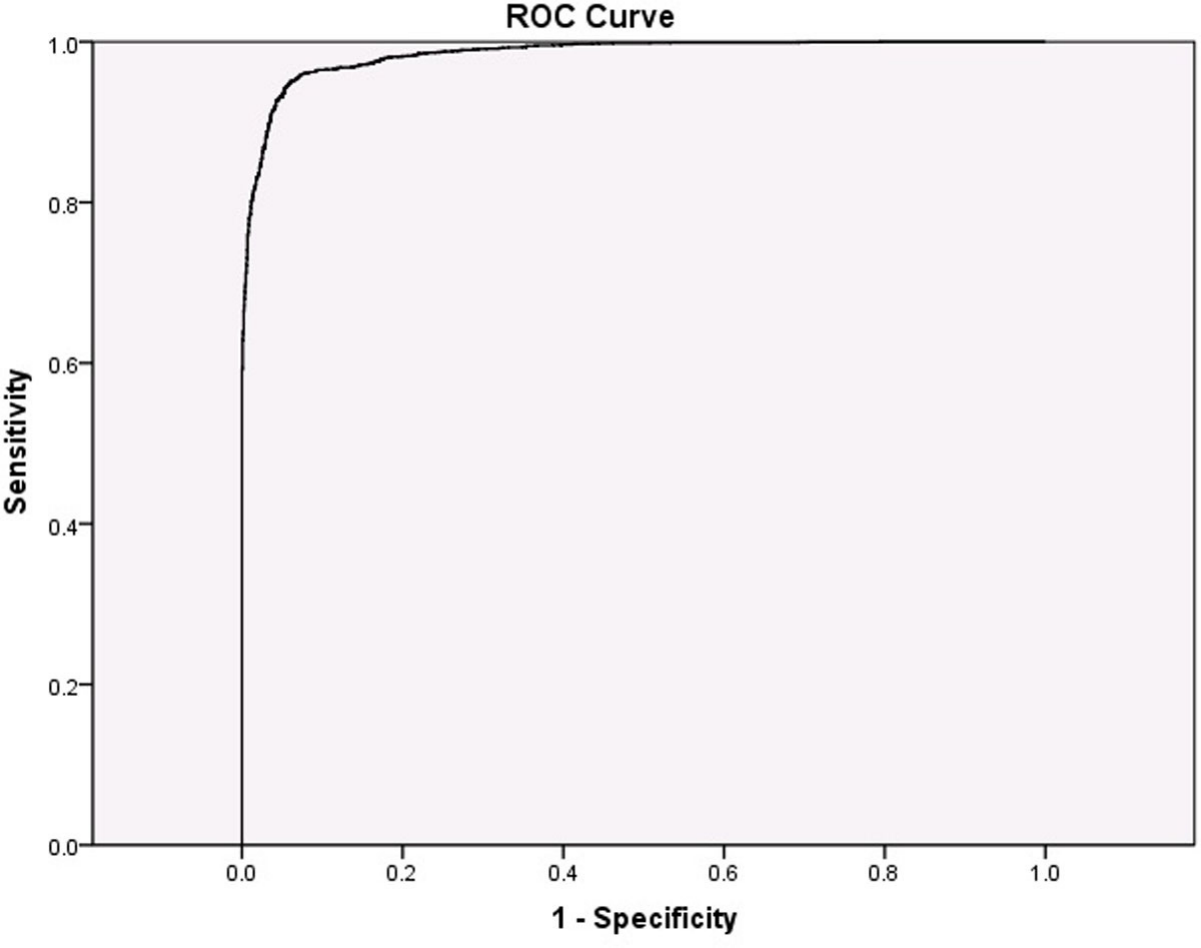
**The ROC curve (Relative Operating Characteristic) and the area under curve for the best hybrid model at 90% redundancy**.

The result of the validation datatsets on six species (on all three models) are shown in Table 6. The hybrid model trained at 90% redundancy had the best accuracy only with exception of *Rhizobium fredii *for which the 50% redundant model was better. As can be seen from Table 5, the hybrid model at 90% redundancy performed best overall for most species. It is possible that the decrease in performance obtained by removing more proteins based on their similarities is not due to the identity value, but due to a resulting imbalance in the training datasets since the redundancy criteria affected proteobacterial protein numbers more strongly than they did the plant protein numbers. Because these estimates are sensitive to the threshold for distinguishing positives from negatives, we constructed a ROC curve to examine the model's accuracy. ROC has been used to show the accuracy of constructed models [21-29]. The ROC curve is a graphical representation of sensitivity (true positive rate) vs. one minus specificity (false positive rate or true negative rate) for any binary classifier system [30]. It is a threshold independent evaluation parameter and gives a value known as Area Under Curve (AUC) (Figure 5) which shows the performance of a classifier in a two class problem [31]. The higher the AUC, the more accurate the model. In the present study the AUC for hybrid model was 0.985 and therefore demonstrated the accuracy of the model.

**Table 6:** Validation (accuracy) percentage by SVM models trained on AAC, DC and hybrid features.

AAC	Serratia marcescens (127)	Acidovorax citrulli (314)	Rhizobium fredii (16)	Solanum lycopersicum (413)	Phaseolus vulgaris (159)	Cucurbita pepo (15)
30	78.74	98.09	75	93.46	94.97	100
50	70.87	96.18	75	94.19	96.23	100
90	70.87	97.77	75	93.46	96.86	100

DC						

30	73.23	96.82	75	94.19	97.48	100
50	75.59	97.45	68.75	94.92	96.84	100
90	74.8	97.77	62.5	95.16	98.72	100

Hybrid (AAC+DC)						

30	74.8	96.5	75	95.4	98.74	100
50	79.53	99.04	81.25	93.46	97.48	100
90	81.1	99.04	79.53	93.95	98.11	100

The accuracy is calculated by dividing the number of correct predictions by total number of protein inputs. The numbers of reviewed proteins are shown in parentheses.

This SVM model can be used to assign a query sequence as to whether it originated from a plant or proteobacterium, thus enabling timely detection of the infection. It may also be used to identify food contamination with bacteria by screening samples by sequencing. SVM models can be used to work in the area of animal proteins. As we have developed a model for plant and proteobacteria, another model can be designed for animal protein and pathogenic proteobacterial proteins. Thus, SVMs can be used in a variety of fields of study.

## Conclusion

The SVM models based on the hybrid approach using both amino acid and dipeptide features exhibited the maximum accuracy on both threshold dependent and threshold independent parameters. Best results were obtained with an RBF kernel and considering protein sets that did not contain any proteins that are more than 90% identical to another protein in the dataset. SVMs have great potential to handle large datasets and thus can be used for sorting proteobacterial sequences from a mixed background, like those found in metagenomic sequence data. As such, an SVM classifier would be a step forward in surveillance techniques for bacteria that lack previously characterized relatives. It may be useful for determining protein sequences obtained from non-sequenced genomes not yet present in Genbank. Other features like domains specific to nitrogen oxidising or fixing bacteria can also be used even to distinguish a pathogenic proteobacterium from a non-pathogenic proteobacterium. This may be used to determine the kinds of bacterial pathogens present in food samples thus improving food security. Human pathogens that are proteobacterial in nature also exist. Specific SVM models can be trained or designed to distinguish them. Thus SVMs hold greater potential for solving a variety of problems in biology.

## List of abbreviations used

SVM: Support Vector Machine; AAC: Amino Acid Composition; DC: Dipeptide Composition; FAO: Food and Agricultural Organization; ROC: Receiver Operating Characteristic; AUC: Area Under The Curve; TP: True Positive; TN: True Negative; FN: False Negative; FP: False Positive.

## Competing interests

The authors declare that they have no competing interests.

## Authors' contributions

RV designed the study and performed the machine learning analysis and drafted the manuscript. UM conceived of the study, and participated in its design and coordination and helped to draft the manuscript. All authors read and approved the final manuscript.
